# Recent developments in intranasal drug delivery of nanomedicines for the treatment of neuropsychiatric disorders

**DOI:** 10.3389/fmed.2024.1463976

**Published:** 2024-09-19

**Authors:** Anglina Kisku, Ambresh Nishad, Saurabh Agrawal, Rishi Paliwal, Ashok Kumar Datusalia, Gaurav Gupta, Sachin Kumar Singh, Kamal Dua, Kunjbihari Sulakhiya

**Affiliations:** ^1^Neuro Pharmacology Research Laboratory (NPRL), Department of Pharmacy, Indira Gandhi National Tribal University, Amarkantak, India; ^2^Nanomedicine and Bioengineering Research Laboratory (NBRL), Department of Pharmacy, Indira Gandhi National Tribal University, Amarkantak, India; ^3^Laboratory of Molecular NeuroTherapeutics, Department of Pharmacology and Toxicology, National Institute of Pharmaceutical Education and Research (NIPER), Raebareli, Uttar Pradesh, India; ^4^Centre for Research Impact & Outcome, Chitkara College of Pharmacy, Chitkara University, Rajpura, India; ^5^Centre of Medical and Bio-allied Health Sciences Research, Ajman University, Ajman, United Arab Emirates; ^6^Uttaranchal Institute of Pharmaceutical Sciences, Uttaranchal University, Dehradun, India; ^7^School of Pharmaceutical Sciences, Lovely Professional University, Phagwara, India; ^8^Faculty of Health, Australian Research Centre in Complementary and Integrative Medicine, University of Technology, Sydney, NSW, Australia; ^9^Discipline of Pharmacy, Graduate School of Health, University of Technology, Sydney, NSW, Australia

**Keywords:** nanoformulation, intranasal drug delivery, phytochemical and synthetic nanoformulation, nanomedicines, neuropsychiatric disorder

## Abstract

Neuropsychiatric disorders are multifaceted syndromes with confounding neurological explanations. It includes anxiety, depression, autism spectrum disorder, attention deficit hyperactivity disorder, schizophrenia, Tourette’s syndrome, delirium, dementia, vascular cognitive impairment, and apathy etc. Globally, these disorders occupy 15% of all diseases. As per the WHO, India has one of the largest populations of people with mental illnesses worldwide. The blood-brain barrier (BBB) makes it extremely difficult to distribute medicine to target cells in the brain tissues. However, it is possible through novel advancements in nanotechnology, molecular biology, and neurosciences. One such cutting-edge delivery method, nose-to-brain (N2B) drug delivery using nanoformulation (NF), overcomes traditional drug formulation and delivery limitations. Later offers more controlled drug release, better bioavailability, improved patient acceptance, reduced biological interference, and circumvention of BBB. When medicines are delivered via the intranasal (IN) route, they enter the nasal cavity and go to the brain via connections between the olfactory and trigeminal nerves and the nasal mucosa in N2B. Delivering phytochemical, bioactive and synthetic NF is being investigated with the N2B delivery strategy. The mucociliary clearance, enzyme degradation, and drug translocations by efflux mechanisms are significant issues associated with N2B delivery. This review article discusses the types of neuropsychiatric disorders and their treatment with plant-derived as well as synthetic drug-loaded NFs administered via the IN-delivery system. In conclusion, this review provided a comprehensive and critical overview of the IN applicability of plant-derived NFs for psychiatric disorders.

## Graphical abstract

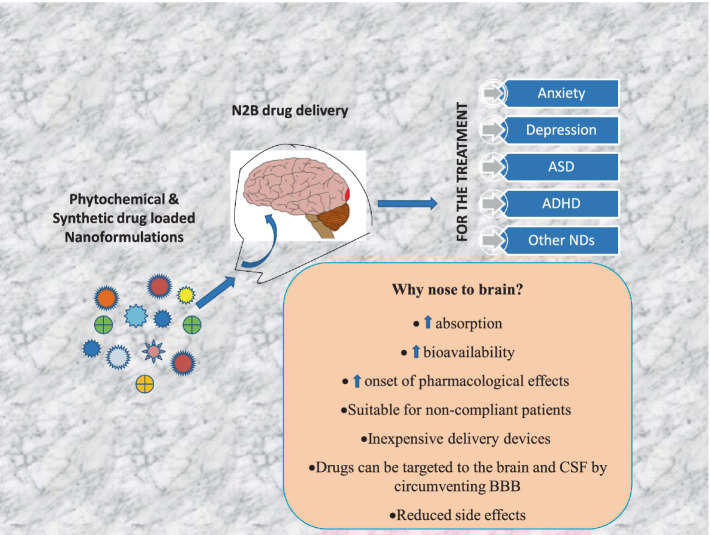

## Introduction

Neuropsychiatric disorders (NPDs) are complex conditions with least understood neurological issues (1). Neuropsychiatric diseases significantly jeopardize the well-being of people affected, wreaking havoc on general health as well as the ability of children and adults to learn and work. Autism, schizophrenia, and bipolar disorder are among the most genetically influenced of many prevalent medical disorders, according to measures of genetic influence such as heritability and recurrence risk ratio (2). Despite the fact that they are thought to represent alterations in brain function, they are not accompanied by evident neuropathology, and the molecular mechanisms behind them remain largely unexplained (1). Nine hundred and seventy million people worldwide, or one in every eight people, experienced some form of mental illness in 2019. Anxiety and depression were the most common mental illnesses. By 2020, there was a significant rise in the number of people experiencing anxiety and despair due to the COVID-19 pandemic. According to preliminary estimates, there has been a 26% increase in anxiety disorders and a 28% increase in severe depressive disorders in just 1 year (3). The authors discovered that mental health and substance use issues were responsible for only 68 per cent of suicides in China, Taiwan, and India after reviewing several meta-analysis studies. According to studies, a substantial majority of suicides are caused by the “dysphoric effect” and “impulsivity” (4). From the 20th century, numerous events sparked the maximum surge of research on mood disorders in the field’s history, surpassing the popularity of psychopathology themes such as schizophrenia and anxiety disorders during those years (2).

Several mental disorders come under NPDs, such as anxiety, depression, autism spectrum disorder (ASD), attention deficit hyperactivity disorder (ADHD) and others like schizophrenia, bipolar disorder, mania, Parkinson’s disease, Tourette’s syndrome, delirium, dementia, vascular cognitive impairment and apathy. The interplay of physiological, genetic, and environmental risk factors contributes to the complex etiology (5). Mental depression has been identified as one of the world’s most serious health issues. According to the monoaminergic (MAO) theory, mental depression is caused by a lack of monoamine (MA) such as norepinephrine (NE), serotonin (5-HT), and dopamine (DA), and medications that increase these monoamine levels are effective antidepressants (6) (Table 1).

**Table 1:** Pathophysiology of neuropsychiatric disorders and clinically available drugs for their treatments.

S. No.	Disease	Pathophysiology	Drugs	Route of administration	Remarks	References
1.	Anxiety	Norepinephrine (NE) and serotonin (5HT) neurotransmitter system abnormalities in anxiety and depression	Alprazolam Chlordiazepoxide Clonazepam Diazepam Doxepine Lorazepam Hydroxyzine Buspirone	ORAL/IV/IM	Less effective	(130, 131)
2.	Depression	Depletion of the neurotransmitter’s serotonin, norepinephrine or dopamine	Ketamine Riluzole Lanicemine Memantine Traxoprodil Rapastinel Dextromethorphan Bupropion Scopolamine Nelivaptan	ORAL/IV/IM	Fail to separate from placebo in terms of efficacy	(130, 132)
3.	Autism spectrum disorder	Although the causes of autism remain unknown, a number of theories, including genetics, immunology, biology, and psychosocial variables, have been put forth. Complex in nature, autism typically has multiple underlying causes that co-occur	Acamprosate Atomoxetine Baclofen DMXB-A (nicotinic agonist) Buspirone Dextromethorphan hydrobromide/quinidine sulfate Divalproex Donepezil EPI-743 Folinic acid Methylphenidate (extended-release) Oxytocin Rapalogues RG7314 (vasopressin V1A receptor antagonist) Riluzole Vitamin D_3_	ORAL/IT/IN	Side effects like significant weight gain, metabolic syndrome, and tachycardia	(132, 133)
4.	ADHD	Specifically, frontostriatal or mesocortical brain network dysfunctions may cause cognitive deficiencies, whereas mesolimbic dopaminergic system dysfunctions may cause issues with reward processing	Methylphenidate immediate release Methylphenidate sustained release Amphetamine liquid Atomoxetine	ORAL	Decreased appetite and sleep disturbances	(134, 135)
5.	Others					
5a.	Schizophrenia	Dysregulation of multiple pathways in Dopaminergic, glutamatergic and GABAergic neurotransmitters	Clozapine Chlorpromazine Fluphenazine Thioridazine Thiothixene Haloperidol Fixed random Butaperazine Olanzapine Quetiapine Risperidone Ziprasidone	ORAL/IM	Possibilities of early relapse	(59, 136)
5b.	Bipolar disorder	Chemical imbalances in the brain	Clozapine Carbamazepine Lithium Risperidone Sertindole Olanzapine Trimipramine	ORAL/IM	Long-term risk of tardive dyskinesia	(46, 137)
5c.	Parkinson’s disease	The gradual loss of cells in the substantia nigra of the brain	Levodopa Carbidopa Pergolide Pramipexole dihydrochloride Ropinirole hydrochloride Rotigotine Apomorphine hydrochloride Selegiline Rasagiline	ORAL/IM	Reduced ability to smell and detect odours	(47, 138)
5d.	Tourette’s syndrome	Disturbance in the cortico-striatal-thalamic-cortical (mesolimbic) circuit	Clonidine Guanfacine Haloperidol Fluphenazine Pimozide Risperidone Tetrabenazine	ORAL/IV/IM	Alpha-agonists cause sedation, dizziness, irritability and headache	(51, 139)
5e.	Panic disorder	Its pathophysiology involves neuroimmune, neurochemical, genetic, and psychological factors. Neuroimmune mechanisms involve dysfunctional body-to-brain communication, with immune and humoral pathways playing a significant role. Genetic factors include polymorphisms in immune and inflammatory responses, particularly in fear-conditioned brain regions. Psychological theories emphasize the fear of fear cycle, with cognitive-behavioral therapies effective in treating panic disorder	Imipramine Alprazolam	ORAL/IM	No fixed-dose method is available	(140)
5f.	Epilepsy	Largely defined by an imbalance in the activity of the excitatory and inhibitory brain regions. Neural circuit dynamics, dendritic spine plasticity, neurotransmitter interactions, and inflammatory processes are some elements that affect this imbalance	Oxcarbazepine Lamotrigine Zonisamide Stiripentol Phenytoin Primidone	ORAL/IM	Slow hepatic metabolism	(60)
5g.	Multiple sclerosis	Environmental variables, autoimmune mechanisms, and genetic predispositions all play a role in the pathophysiology of multiple sclerosis. The illness appears through a range of symptoms because to the semi-random distribution of lesions in the CNS, which can lead to both loss of function and positive feelings	Interferon beta Glatiramer acetate Teriflunomide Dimethyl fumarate Fingolimod Natalizumab Alemtuzumab Mitoxantrone	ORAL/IM	Do not cure the disease or reverse the damage that has occurred with prior events	(61)
5h.	Pick’s disease	The pathological hallmark of Pick’s illness is the build-up of 3-repeat tau protein in the form of spherical, para-nuclear neuronal aggregates called Pick bodies	Cholestyramine DMSO Lovastatin Nicotinic acid	ORAL/IV/IM	Non-uniform clinical data	(62)
5i.	Huntington’s disease	Huntington’s disease (HD) is a fatal neurodegenerative disorder caused by a polyglutamine expansion in the huntingtin gene, leading to motor, cognitive, and psychiatric impairments	Sulpiride Haloperidol Tetrabenazine Lamotrigine Donepezil Tetrabenazine Fluoxetine Thiopropazate	ORAL/IV/IM	Long-term use can produce parkinsonism, akathisia, acute dystonias, tardive dyskinesia	(49)
5j.	Narcolepsy	The disorder is predominantly associated with the loss of orexin (hypocretin) neurons in the hypothalamus, which play a crucial role in maintaining wakefulness and regulating sleep-wake transitions	Modafinil Armodafinil Methylphenidate Pitolisant Sodium oxybate Amphetamines Mazindol Sodium oxybate Venlafaxine Clomipramine	ORAL/IV/IM	Different peptides do not readily cross the blood-brain barrier	(63)
	Wilson’s disease	The pathophysiology involves complex interactions between subcortical dysfunction, disrupted brain network connectivity, neurovascular coupling imbalances, and neuroinflammation	Zinc acetate Trientine D-Penicillamine Tetrathiomolybdate	ORAL/IV/IM	Higher risk of liver injury	(64)
	Prion diseases	The pathophysiology of prion diseases involves a combination of redox imbalance, misfolding of the prion protein (PrPC) into a pathogenic form (PrPSc), synaptic dysfunction, and transcriptional dysregulation	Suramin Curcumin Quinacrine Chlorpromazine Quinine and biquinoline Mefloquine	ORAL/IV/IM	Cannot provide reversal of symptoms or significant prolongation of survival	(50)

Conventional dosage forms such as tablets, capsules, powders, mixtures, injections, and transdermal patches have several challenges, such as poor bioavailability and fluctuations of drugs in plasma (7). Therefore, novel dosage forms and drug delivery systems can be opted to overcome poor bioavailability, limited absorption (food effect), first-pass metabolism, drug toxicity, and dose-dependent adverse effects (6). The difficulties in early diagnosis, the long disease courses, and the difficulties of drug delivery in overcoming biological barriers have prevented the therapeutics for treating psychological disorders and mental illness from being successful to date (blood-brain barrier). A potential tool for accurate diagnosis, effective prescription, precise imaging, and distinct psychiatric diseases has recently been identified as nanotherapeutic. Nanotechnology has developed new approaches to innovate specific therapeutics for treating neurological and psychiatric diseases in response to paradigmatic crises (8). Nano-therapy refers to systems that are 10–100 nanometers in size and have unique physiological and chemical features. Plasticity and changed surface qualities, flexibility, and controlled release of the active medicinal ingredient are all characteristics of nano-preparations. Nano-therapeutics provide active medications to the brain that are specifically tailored for the central nervous system (CNS) (8).

Intranasal (IN) drug delivery has proven to be a significant benefit over other routes of drug administration due to enhanced bioavailability in the brain. Because the BBB prevents various medications from reaching the brain, IN drug administration has overcome this problem. The BBB protects the brain’s equilibrium by preventing most outside substances, such as lipids, peptides, and vital nutrients, from entering the brain. As a result, practically all medications and bioactive have difficulty entering the brain (9). Moreover, the NFs, distinguished by their small size and capacity to penetrate the BBB, are one of the promising ways to overcome the BBB. This form of drug delivery dramatically improves the bioavailability of drugs while reducing adverse effects. As a result, general approaches for improving medication transport to the brain are of enormous pharmacological interest. Non-invasive methods are widely used, encompassing chemical drug delivery, carrier-mediated drug delivery, receptor/vector-mediated drug delivery, lipophilic analogue synthesis, prodrugs, and IN drug delivery, which employs the olfactory and trigeminal neural pathways to deliver drugs to the brain. N2B targeting does have some drawbacks: IN insulin (Nasulin^®^) did not substitute for subcutaneous insulin injections because precise dosing of intranasally applied drugs is still a problem that needs to be resolved (10). This paper focuses on the latest information on possible nano-therapy applications via the IN route in the treatment of mental and neurological disorders such as anxiety, depression, autism spectrum disorders (ASDs), attention deficit hyperactivity disorders (ADHDs) and others. It also provides information regarding medicinal plants and their NFs, which have psychoactive qualities that have been utilized to treat neuropsychiatric disorders.

### Anxiety

Anxiety is a perfectly natural feeling in which the brain responds to stress and provides warning toward impending danger. Anxiety disorders may be of different types, such as generalized anxiety disorder, agoraphobia, panic disorder, social anxiety disorder, selective mutism, and particular phobias. These prevalent and problematic ailments often begin in childhood, adolescence, and early adulthood (11). Avoidance behavior is most likely to develop when anxiety becomes too frequent, intense, and persistent (12). The occurrence of anxiety in women is two times more as compared to men. Worldwide, the prevalence rate is 0.28% for post-traumatic stress disorder (PTSD), 1.1% for obsessive-compulsive disorder (OCD), and 0.4% for panic disorder (13). Although developments in neurobiology and treatments have focused emphasis on how anxiety disorders are classified, the categorization of these diseases has not altered drastically over several decades (14). The *DSM-5* process and the resulting publication of *DSM-5* in 2013 have had a considerable impact on the classification of anxiety disorders (15).

Anxiety is significantly impacted by stress. The altered physiological and psychological reactions that affect various organs and systems, including the central nervous system (CNS), as well as the increased vulnerability to various psychiatric diseases, including depression, are a result of stressful events (16). According to animal models of anxiety disorders, anxiety is one of the most prevalent psychological reactions in patients awaiting surgery, with up to the hypothalamic-pituitary-adrenal (HPA) axis and stress-responsive brain areas (17). Anxiety disorders are the result of a complicated interaction of biological, psychological, temperamental, and environmental factors. According to a growing body of research, the perception of noxious stimuli activates a “threat circuit” in the brain, consisting of bilateral connections between the dorsomedial prefrontal cortex, insula, and amygdala. Changes in the HPA axis appear to be state-dependent, and they tend to improve once the anxiety syndrome is resolved (17). They can occur in conjunction with other mental health conditions, such as depression. Recent research has focused on the participatory character of contemporary media, particularly social media, and how it affects anxiety and depression (18).

Anxiety disorders are documented and categorized in diagnostic systems such as the American Psychiatric Association’s Diagnostic and Statistical Manual of Mental Disorders (DSM, now version IV-TR) or the International Classification of Diseases (ICD) (ICD, current version 10, World Health Organization) (19). Cognitive-behavioral therapy and medicines (SSRIs, SNRIs, benzodiazepines, and azapirones) are two evidence-based treatments for anxiety disorders. Serotonin plays a vital role in anxiety, which inhibits the circuit responsible for the threat response. The neuronal activity in the dorsomedial prefrontal cortex–amygdala circuit to aversive stimuli is reduced by selective serotonin reuptake inhibitors (SSRIs), which are first-line therapies for a range of anxiety disorders (20). Benzodiazepines and their receptors are used to develop various anxiolytic medicines (second-generation anxiolytics). Although there is no consensus on the next steps for treatment-resistant anxiety disorders, therapeutic options include a combination of medications. Add-on augmentation techniques such as pregabalin, gabapentin, and quetiapine, as well as cognitive-behavioral therapy and antidepressant medication, are used clinically (20).

Anxiety and its sequelae, if left untreated, can have a substantial impact on a child’s life and can follow them into adulthood (21). These uncomfortable feelings can be effectively treated with behavioral strategies such as cognitive behavioral therapy and pharmaceutical medications (22). Even though SSRIs are more helpful than psychotherapy in the treatment of late-life anxiety, many elderly anxious people prefer psychosocial therapies (23). Although pharmacological and psychological therapies can help some people, they aren’t beneficial for everyone and aren’t enough to address prevalent physical health issues like an increased risk of cardiovascular disease (CVD) (24).

### Depression

Depression is a most commonly occurring NPD, which is characterized by persistent sadness or irritated mood, anhedonia, worthlessness, hopelessness, insomnia, and physical and cognitive alterations that have a major impact on a person’s ability to function (8, 9, 25). Further, it leads to severe suffering, morbidity, mortality, and financial loss. It affects people of all ages and leads to suicide if untreated (10). Currently, it is the third leading cause of death and is expected to become the first cause of death by 2030 (26). In India, it is the most commonly occurring mental condition, which affects around 45.7 million people (12). However, the condition is frequently misdiagnosed, and physicians have difficulties providing the proper treatments due to the complexity of the pathophysiology (12). The pathophysiology of depression involves modifications to multiple pathways, such as the hyperactivity of the hypothalamic-pituitary-adrenal (HPA) axis, dysregulation of the oxidant/antioxidant system balance, increased levels of inflammatory cytokines, leptin resistance, altered plasma glucose, insulin resistance, neuronal brain-derived neurotrophic factor (BDNF) reduction, and decreased serotonergic neurotransmission in different brain regions (27). Findings of earlier research have shown that stresses, socioeconomic conditions such as poverty, un-hygienic lifestyle, parental depression, and infections are the risk factors for the development of depression (2). Addison’s illness, Cushing syndrome, hypothyroidism, and hypogonadism are all examples of syndromal hormonal changes that can cause depressive symptoms. When depressed symptoms coexist with subclinical changes in hormonal axes, it’s difficult to say whether the subclinical changes produce depressive symptoms or are present independently (28). On the other hand, epigenetic pathways may mediate the long-term increases in depression risk associated with unfavorable life events, as well as provide a mechanistic foundation for integrating genetic and environmental components (29). For unipolar depression, posttraumatic stress disorder, and treatment-resistant depression, exercise and yoga seem to be the most successful supplemental therapies. For depression, mindfulness-based meditation works well as a stand-alone treatment or as an adjuvant, with results that last up to 6 months (30).

Sleep issues are no longer an inevitable consequence of depression but rather a predictive paroxysmal symptom, according to the bidirectional relationship between sleep disturbance and depression. Antidepressants can exacerbate sleep disturbances, and hypnotics can cause depression. Sleeplessness may alter the behavior of the individual, which ultimately can lead to depression and anxiety (31). Riluzole may help treat unipolar depression in two open-label studies that support this claim, and a third open-label add-on study found improvements in bipolar depression. In two placebo-controlled trials, a single subanesthetic dosage of intravenous ketamine (0.5 mg/kg infusion over 40 min) was used to treat unipolar depression. The results showed rapid but temporary antidepressant efficacy. Acute euphoric and psychotomimetic side effects were noticed (2–4 h after intravenous administration). Still, they occurred at a different time than the improvement of the core depressive symptoms, which in some cases persisted for up to a week (32).

### Autism spectrum disorder

A prevalent neurodevelopmental illness known as autism spectrum disorder (ASD) is typified by repetitive behaviors and disrupted social interaction. ASD has a solid genetic component, and around 1,000 genes are thought to be involved in its pathogenesis (31). It is challenging to identify ASD symptoms at its beginning, posing a potential threat to the child in adulthood (32). In the majority of ASD cases, the contributing genetic alleles have not been identified. Even though hundreds of ASD risk genes have been discovered, each gene’s contribution to the ASD population is very tiny, with none detected in more than 2% of patients. Because the efficacy of medications for treating the core symptoms of autism has not been proven, they are mainly used to treat related symptoms of autism spectrum disorder. Anxiety, hyperactivity, impulsivity, aggression, insomnia, and irritability are some of the symptoms that are specifically linked to the condition. Although the majority of experts currently agree that a central nervous system disorder causes autism, there are differing perspectives on its defining traits and the causal chain that connects brain dysfunction to behavioral characteristics (33). Assessment techniques include parent/caregiver interviews, patient interviews, direct patient observation, and comprehensive clinical assessments that entail a detailed examination of a family history for ASD or other neurological issues (34).

Risperidone and aripiprazole have been licensed by the Food and Drug Administration (FDA) to treat irritability linked to an autism spectrum disorder diagnosis. Risperidone is approved for children over the age of five, whereas aripiprazole is approved for children over the age of six (35). Different classes of drugs used in the treatment of ASD like atypical antipsychotics, selective serotonin reuptake inhibitors (SSRI), tricyclic antidepressants, anticonvulsants, opiate antagonists, psychostimulants, ACE inhibitors, alpha-2 receptor agonists and glutamate antagonists (36).

### Attention deficit hyperactivity disorder

Attention deficit-hyperactivity disorder (ADHD) manifests through symptoms of hyperactivity and impulsivity, inattention, or a blend of hyperactivity, impulsivity, and inattention that deviates from the expected developmental stage and hinders daily functioning. The disorder is commonly diagnosed in children, and symptoms that lead to impairment in functioning can last into adulthood in up to 70% of cases (37). The reported prevalence of attention-deficit/hyperactivity disorder (ADHD) stands at 9.40% in male children and 5.20% in female children, with a prevalence range of 7.6 to 15% in children aged between 8 and 15 years. The prevalence of ADHD in Indian children aligns with the global prevalence rates (38). ADHD has a significant genetic component, with heritability ranging from 0.75 to 0.91. Genetic studies have focused primarily on candidate genes involved in dopaminergic transmission for various reasons. Its symptoms that persist into adolescence are associated with more excellent academic, behavioral, and social impairment. Adults with persistent symptoms have less formal education, lower-status jobs (but the same rates of employment), and higher rates of antisocial personality (39). The etiology of ADHD is undeniably influenced by both genetic and environmental factors, resulting in alterations to the maturing brain and yielding a diverse array of neuropsychological, structural, and functional irregularities (40). Dopaminergic drugs are clinically effective; imaging studies have linked front-striatal circuitry (rich in dopaminergic innervation) to ADHD.

Comorbidity is a common clinical feature in ADHD patients throughout most of their lives. Oppositional defiant disorder, conduct disorder, mood disorders (unipolar and bipolar), anxiety disorders, and learning difficulties are among the mental illnesses linked to ADHD in children. In a systematic study of the impact of gender on the clinical characteristics of ADHD, Biederman and team discovered that girls with ADHD had a lower probability of comorbid disruptive behavior disorder than boys with ADHD (41, 42). The most effective treatment for ADHD in adults combines pharmacologic and educational measures. Dopaminergic drugs are clinically effective; imaging studies have linked front-striatal circuitry (rich in dopaminergic innervation) to ADHD (39). Stimulants such as dextroamphetamine, methamphetamine, methylphenidate, and pemoline are the most effective ADHD treatments. When given methylphenidate or amphetamines, approximately 70% of patients improve moderately to significantly. ADHD is a common and, until recently, almost always unnoticed diagnosis. Its identification is contingent on obtaining an accurate childhood psychiatric diagnosis (43).

### Other disorders

Some of the other disorders that fall under the category of NPDs are Schizophrenia, epilepsy, bipolar disorder, Parkinson’s disease, mania, multiple sclerosis, Pick’s disease, Huntington’s disease, Wilson’s disease, Prion disease, Tourette’s syndrome, delirium, dementia, vascular cognitive impairment, and apathy. Some of the neuropsychiatric disorders show respective pathology in Schizophrenia; dysregulation of multiple pathways in Dopaminergic, glutamatergic and GABAergic neurotransmitters can be seen. Early schizophrenia patients who received methotrexate treatment saw a selective improvement in their positive symptoms, with no adverse effects on their negative symptoms or cognitive function (44). Around 45 million people worldwide are affected by bipolar disorder. It usually includes both manic and depressive episodes, with periods of everyday mood in between (45). Bipolar disorder results due to chemical imbalances in the brain. Clozapine, carbamazepine, lithium, risperidone, sertindole, olanzapine and trimipramine are the currently used drugs for the treatment of bipolar disorder (46). Parkinson’s disease results from the gradual breakdown of cells in the substantia nigra area of the brain. Medications utilized in its management include levodopa, carbidopa, pergolide, pramipexole dihydrochloride, ropinirole hydrochloride, rotigotine, apomorphine hydrochloride, selegiline, and rasagiline (47). The start of mania can be connected to raised stress levels, disturbances in sleep patterns, or sleep deprivation. Multiple sclerosis forms when immune cells are activated and penetrate the central nervous system, causing inflammation. Treatment alternatives feature interferon-beta, glatiramer acetate, teriflunomide, dimethyl fumarate, fingolimod, natalizumab, alemtuzumab, and mitoxantrone (48). Pick’s disease is characterized by significant loss of large pyramidal neurons in the cortex and cytoarchitectural changes, diffuse spongiosis, and gliosis. Cholestyramine, DMSO, lovastatin, and nicotinic acid are among the pharmacological interventions employed. Huntington’s illness is a familial neurodegenerative disorder resulting from an unstable expansion of a CAG repeat. Currently used drugs for the treatment are sulpiride, haloperidol, tetrabenazine, lamotrigine, donepezil, tetrabenazine, fluoxetine, and thiopropazate (49). Wilson’s disease is caused by the deletion of genetic material from a specific region of the chromosome. Zinc acetate, trientine, D-penicillamine, and tetrathiomolybdate are used for the treatment. When a single gene is expressed, a single prion protein (PrP) is produced, which can then be transformed into the only disease-causing factor, a misfolded prion. Drugs used for the treatment are suramin, curcumin, quinacrine, chlorpromazine, quinine and quinoline, and mefloquine (50). Tourette’s syndrome shows disturbance in the cortico-striatal-thalamic-cortical (mesolimbic) circuit. A decrease in the brain’s oxidative metabolism during delirium results in cerebral dysfunction because of anomalies in several neurotransmitter systems.

First-line treatments are clonidine, guanfacine, haloperidol, fluphenazine, pimozide, risperidone, and tetrabenazine (51). Instead of 5-HT2A antagonism or 5-HT1AR partial agonism, 5-HT7R antagonism is a critical factor in lurasidone’s effects on cognitive function when used to treat atypical antipsychotic-resistant cognitive impairment. One of the side effects of lurasidone that requires attention, along with the 5-HT7R downregulation brought on by long-term administration, is the disruption of sleep (52).

Apathy is a complex symptom involving emotional, behavioral, and cognitive aspects, such as decreased goal-directed activity and motivation. All the currently used drugs for the treatment of neuropsychiatric diseases are mentioned in Table 1.

## Challenges in drug delivery for neuropsychiatric disorders

The delivery of medication for neuropsychiatric conditions encounters substantial difficulties, mainly because of the safeguarding features of the blood-brain barrier (BBB) and the complicated biological mechanisms involved in these disorders. In light of the advancements in pharmacological treatments, securing effective care remains a continual hurdle, requiring the innovation of fresh techniques to better facilitate drug access to the central nervous system (CNS). This discourse examines the obstacles and prospective resolutions pertaining to drug delivery for neuropsychiatric disorders, particularly emphasizing the contributions of nanotechnology and other sophisticated delivery systems.

The BBB acts as a fundamental safeguard against the distribution of drugs in the CNS, hindering the influx of numerous therapeutic elements, including antibiotics and neuropeptides, into brain matter. This barrier serves an essential role in safeguarding the brain from deleterious substances, yet it complicates the therapeutic management of neuropsychiatric disorders (53, 54). Conventional pharmacological agents frequently fail to achieve therapeutic concentrations within the brain due to the restrictive nature of the BBB, thereby necessitating elevated dosages that may precipitate systemic side effects and diminish patient adherence (55, 56).

Nanotechnology posits promising strategies for surmounting the challenges posed by the BBB. Polymeric nanoforms, lipid-derived transporters, and nanoemulsion systems, categorized as nanocarriers, could greatly benefit drug delivery by enhancing their ability to traverse the BBB and improving the stability and bioavailability of therapeutic compounds (8, 57). Lipid NFs, specifically, have demonstrated efficacy in facilitating the transport of antipsychotics across the BBB, enabling controlled and targeted drug release whilst mitigating adverse effects (55). The approach of administering nanosystems through the nasal cavity is gaining attention as a viable option, facilitating direct transfer to the brain using neural pathways, which reduces systemic side effects while improving therapeutic results (56, 58).

Gene therapy, enabled through the utilization of nanoparticles, signifies an avant-garde approach for the management of psychiatric disorders. Nanoparticles can be meticulously engineered to convey genetic material across cellular barriers, thereby potentially addressing the genetic foundations of these disorders. Despite the promise inherent in gene therapy, there exist substantial challenges in guaranteeing safe and efficacious delivery, thus necessitating further investigative efforts to translate these methodologies into clinical applications (59).

Although nanotechnology-based platforms exhibit considerable potential, their translation into clinical practice encounters various obstacles, including regulatory complexities, scalability issues, and concerns regarding long-term safety (57, 58). A greater volume of preclinical and clinical investigations is imperative to establish the efficacy and safety profiles of these novel delivery systems. Combining nanotechnology with standard therapeutic approaches may greatly improve how medications work and are administered, potentially transforming the care for neuropsychiatric issues (57).

The process of developing new drugs has been made more difficult by the morphing diagnostic boundaries of anxiety disorders and the lack of reliable, valid human biomarkers. Several experimental drugs fall short of the placebo’s effectiveness (54). However, a standard therapy study arm is rarely used in studies (55) Clozapine’s use in ASD has been constrained by side effects like significant weight gain, metabolic syndrome, and tachycardia. In adults with ASD, standard fluoxetine reduced repetitive behaviours (obsessive-compulsive symptoms). Still, a melt-in-your-mouth version of the medication had no more significant impact on child symptoms than a placebo. Children with ASD were found to have poorer cognition after taking memantine (56). Psychostimulant therapy is used to treat ADHD, but the most common side effects include decreased appetite and sleep disturbances (57). It is challenging to assess the relationship between imipramine or alprazolam dose (or plasma level) and clinical response in panic disorders because there is no fixed-dose method available (58). It is necessary to conduct more research to assess anecdotal claims that SSRIs and SNRIs cause more seizures in patients with slow hepatic metabolism or when taken in excess (60). How intricate brain networks contribute to mental illnesses is difficult to quantify. Targets part of complicated networks may also have adverse effects, and high-affinity compounds, particularly smaller molecules, may bind off-target and have toxic effects. Both the pathophysiology and origin of the disease are complicated and poorly understood. It keeps making it more challenging to find suitable pharmacological targets. Firmly target-specific drug candidate molecules may be less effective in complex brain networks or have side effects restricting their use. The translational value of animal models used in drug development studies is minimal. An issue contributing to new drug candidates’ lack of efficacy in clinical trials is an over-reliance on mouse behavioural models in drug development research. Clinical trial designs are essential for moving drug candidates through the research process, choosing approved product labels, and ultimately using the product to treat diseases. Not least of all, medication non-adherence is a significant barrier to effective therapy. Medication non-adherence in mental disorders is caused by a variety of factors, including a lack of understanding, therapeutic beliefs, drug abuse, adverse drug reactions, and the recurrence of positive symptoms.

## Nose-to-brain drug delivery

**Figure 1:**
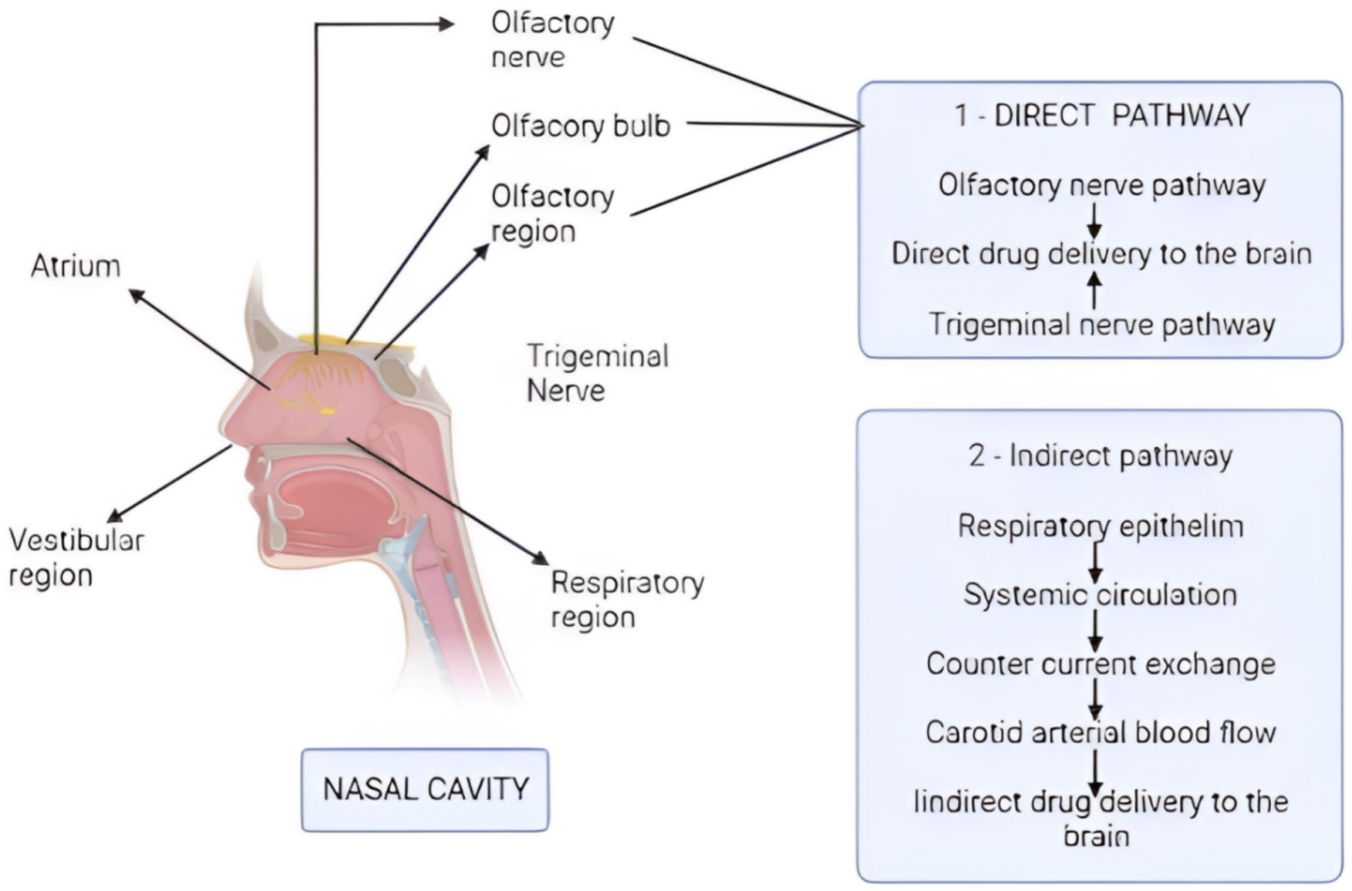
Mechanism of drug absorption via direct and indirect pathways. The direct pathway starts from the olfactory and trigeminal nerves, which deliver the drug directly to the brain. The indirect pathway starts from the respiratory epithelium, and the drug reaches to the carotid arterial blood, thus providing the medicines to the brain indirectly. (Created with BioRender.com).

Nasal drug delivery methods necessitate a comprehensive comprehension of the nasal cavity’s anatomy and physiology. The nasal cavity comprises the vestibule, respiratory, and olfactory regions (see Figure 1). The respiratory region, densely populated with blood capillaries, facilitates systemic drug absorption and subsequent indirect drug transport to the IN route (61). Olfactory nerves directly connect the olfactory region’s upper part and the brain, specifically the frontal cortex and olfactory bulb (9). A profound understanding of the nasal cavity’s function, anatomical structure, and cellular composition is crucial for investigating nasal drug absorption and the molecular pathways to the brain. Mucus can ensnare various molecules, transporting them to the throat for absorption into the GI tract. Given the limited absorption capacity, a drug’s solubility and potency typically serve as limiting factors. Direct absorption from the nasal cavity to the brain notably bypasses pre-absorption metabolism, first-pass effects, and dilution resulting from distribution and protein binding (62). Consequently, drugs must traverse the mucus layer to access the epithelium’s surface for absorption (61). The primary objective of these drug delivery routes is to achieve the desired drug concentrations at the target site while potentially slowing drug degradation and reducing physical clearance (61).

**Figure 2:**
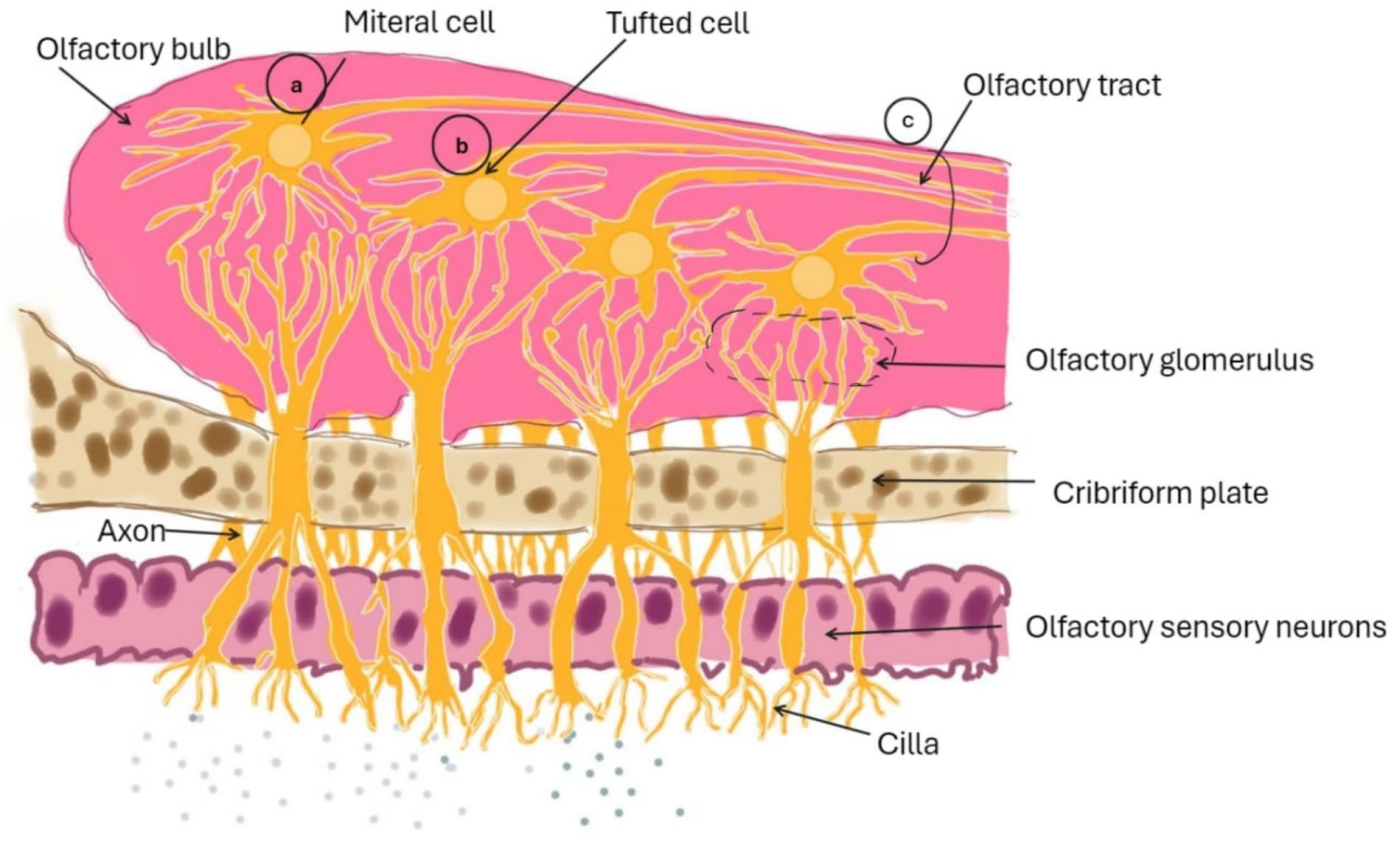
Diagram illustrating the medication transport route from the N2B via intracellular olfactory pathway.

Intracellular transport, extracellular transport, and a combination thereof mediate direct drug delivery from the N2B. The intracellular transport-mediated route facilitates the arrival of intranasally administered drugs to the olfactory bulb after several hours. The two extracellular transport-mediated mechanisms can explain the swift entry of medicines into the brain post-IN delivery (1). Both direct and indirect pathways can penetrate the blood–brain barrier (BBB), as illustrated in Figure 2.

**Figure 3:**
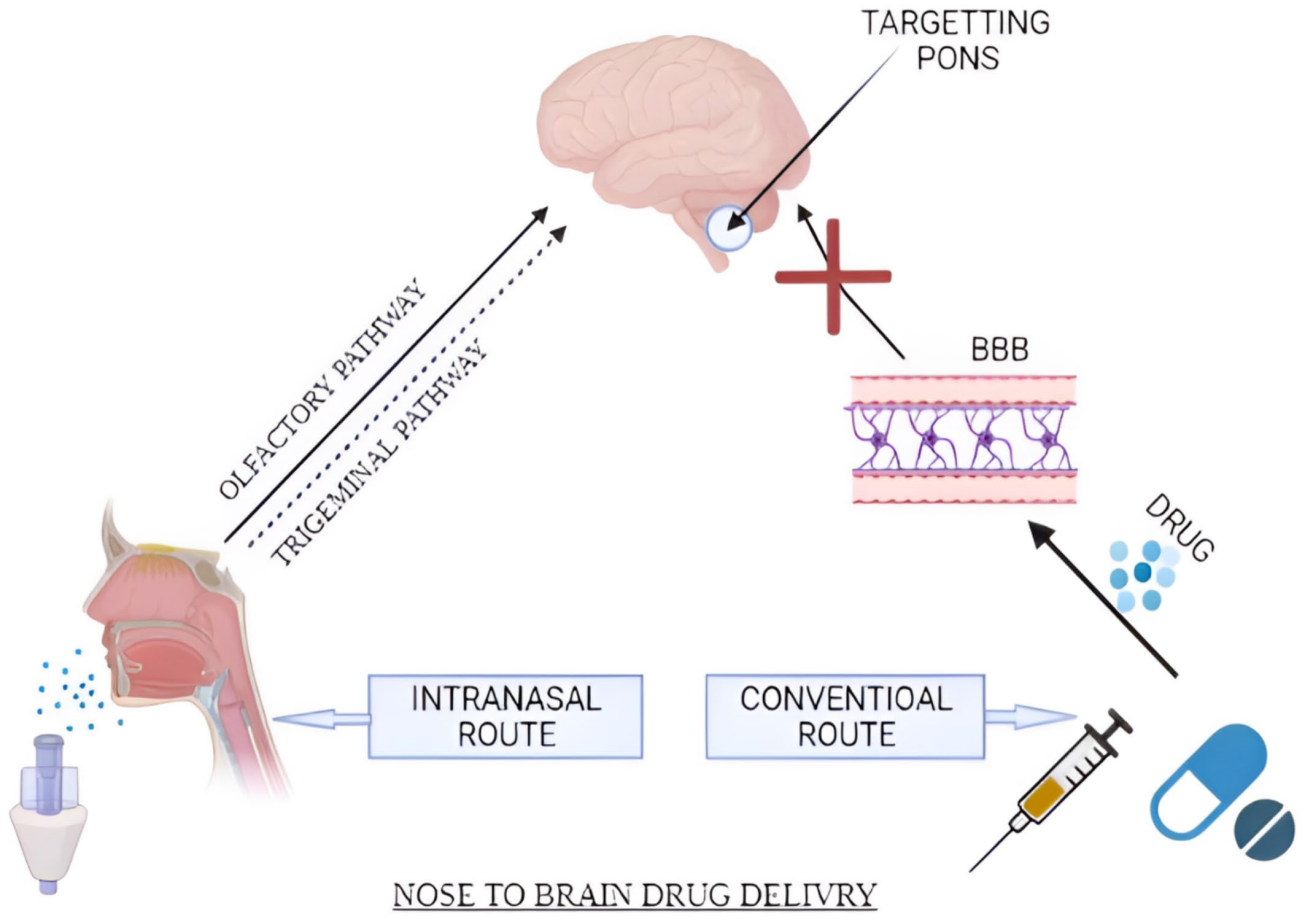
Schematic diagram representing IN and conventional routes of the drug. Drug transportation occurs via major (olfactory and trigeminal pathways) and minor (systemic circulation pathways). (Created with BioRender.com).

Figure 3 presents the pathways facilitating drug movement from the nasal cavity to the central nervous system (CNS) through olfactory neurons and ancillary cells. The trigeminal nerve carries sensory signals from the nasal cavity, oral cavity, eyelids, and cornea to the central nervous system, utilising its three divisions: ophthalmic (V1), maxillary (V2), and mandibular (V3) (62). Nerve branches stemming from the ophthalmic section of the trigeminal nerve serve the nasal mucosa’s dorsal region and the nose’s front part, in contrast to those from the maxillary section, which innervate the side areas of the nasal mucosa. The mandibular section of the trigeminal nerve serves the lower jaw and dentition without any neural pathways connecting to the nasal cavity. The three divisions of the trigeminal nerve converge at the trigeminal ganglion and project centrally to penetrate the brain at the level of the pons, concluding in the spinal trigeminal nuclei located within the brainstem (62).

**Figure 4:**
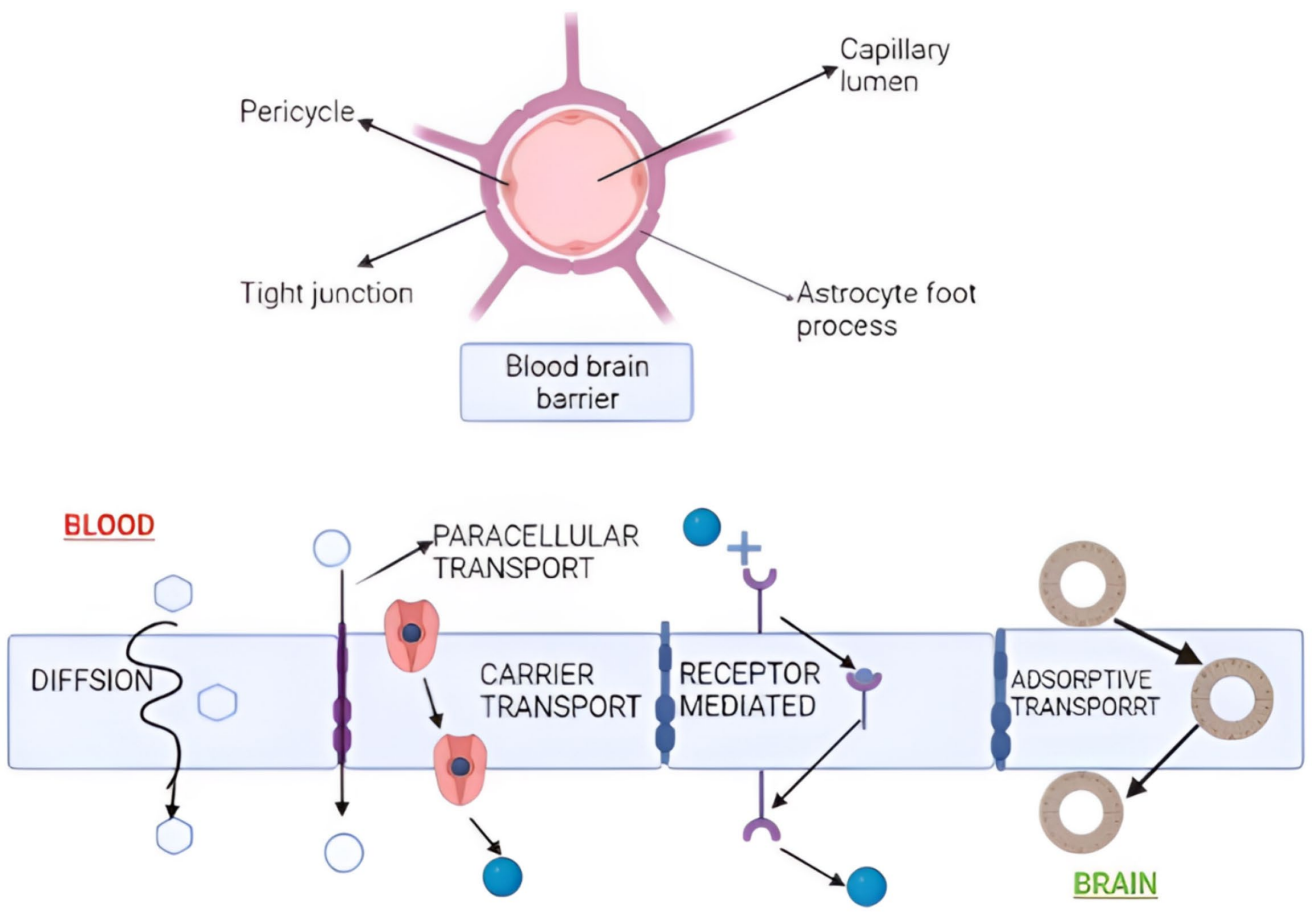
Nanoparticle transportation across the blood-brain barrier. Transportation occurs by diffusion, paracellular, carrier, receptor-mediated, and adsorptive transport. (Created with BioRender.com).

While *in vivo* investigations are the most important for any nasal drug absorption and permeation tests, *in vitro* research can better understand and regulate molecular elements of nasal absorption and drug transport. Intranasal drug administration has been used to treat migraines, acute and chronic pain, Parkinson’s disease, cognitive impairments, autism, schizophrenia, social phobia, and depression, among other things (63). By transporting extracellular fluid, sending immune cells to injury areas, and bypassing first-pass metabolism, the lymphatic system is critical for maintaining homeostasis (64). In comparison to alternative routes of administration, IN drug delivery is considered non-invasive. It offers advantages such as quick drug absorption, rapid onset of action, evasion of first-pass metabolism, and minimal systemic dilution. The IN method bypasses the BBB and delivers the drug straight to the brain, decreasing non-target distribution. Several ways have been presented to demonstrate medication absorption into the brain via the nose. The paracellular and transcellular pathways, on the other hand, are primarily considered. Lipophilic substances easily permeate the nasal mucosa by partitioning into the lipid (bilayer) of the cell membrane and diffusing into the cytoplasm via the transcellular pathway (65). Nasal absorption and permeability are primarily measured using two cell lines (RPMI 2650 and CaCo-2). Previous reports showed that mucoadhesive compounds like pectin and chitosan efficiently extended residence durations at the olfactory epithelium. Mucoadhesive and viscosity-increasing compounds have also been found to boost the bioavailability of nasal formulations intended for systemic distribution. Charlton and the group found that mucoadhesive compounds like pectin and chitosan efficiently extended residence durations at the olfactory epithelium. Mucoadhesive and viscosity-increasing compounds have also been found to boost the bioavailability of nasal formulations intended for systemic distribution. The BBB is a fragile network of blood arteries separated from the circulatory system by firmly packed endothelial cells. It guards the brain against introducing undesirable or dangerous substances like chemicals and toxins (Figure 4). Nasal administration offers various advantages compared to alternative routes. These include non-invasiveness, ease of application, and painlessness compared to parenteral and rectal routes. Additionally, it enables the direct passage of drugs to the bloodstream, circumventing gastrointestinal tract inactivation and rapid hepatic metabolism commonly seen with some oral medications. The vast surface area, permeability, and vascularization of the nasal mucosa contribute to high systemic drug absorption, resulting in a rapid onset of action and enhanced bioavailability compared to oral medications. Besides, pharmaceuticals may be sent directly to the brain using the nasal path, removing the need to circumvent the blood-brain barrier (BBB) (66).

By leveraging the direct connection between olfactory receptor cells and the environment and the central nervous system (CNS), nasal drug delivery enables the evasion of the BBB and promotes direct transfer to the CNS. Extending the residence time in the nasal cavity may prolong drug absorption through the nasal mucosa, potentially enhancing therapeutic outcomes (67). N2B transport has long relied on nanoparticles. Several authors coated the surface of nanoparticles with substances that have a specific affinity for sugar molecules or other components of the nasal mucosa, resulting in increased molecular transport from the N2B. Certain nasal products with possible central nervous system side effects would benefit from inhibiting transit from the N2B. Medicines absorbed through the respiratory area can also reach the brain by being systemically absorbed and passing the BBB. In addition, trigeminal nerves innervate the pulmonary region, providing a route for medications to be delivered directly to the brain, as shown in Figure 2 (68).

The BBB protects the brain’s equilibrium by preventing most outside substances, such as lipids, peptides, and vital nutrients, from entering. As a result, practically all medications and bioactive agents have difficulty entering the brain. The IN route provides a non-invasive, direct route that bypasses the BBB, enhances drug concentration in the brain, lowers systemic adverse effects, is painless, and improves drug performance (9).

**Figure 5:**
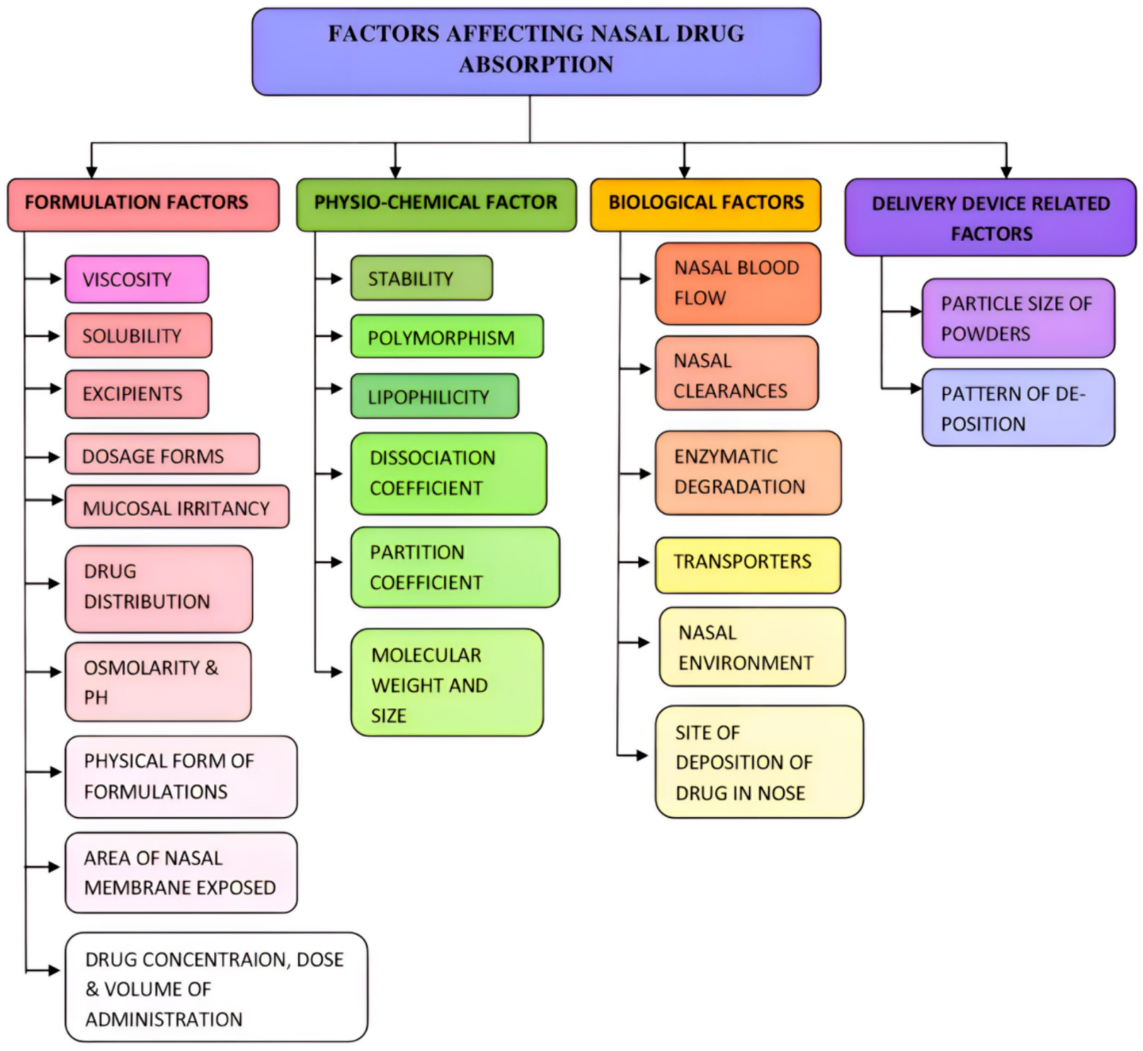
Factors affecting the nasal drug absorption. Various formulations, physio-chemical, biological, and deliver device-related factors affect nasal drug absorption.

Hydrophilic compounds, charged molecules, proteins, and peptides cannot pass through the endothelium cells. However, lipophilic medications, including antidepressants, anxiolytics, and many hormones, can pass (62). Models of nasal drug delivery are employed in the identification and evaluation of drug absorption and permeation, as well as in conducting pharmacokinetic/pharmacodynamic (PK/PD) studies, toxicological assessments, electrophysiology studies, and the evaluation of drug transfer interactions and nasal barrier integrity (62). Various factors should be considered while delivering the drugs through the IN route. Factors affecting drug absorption from IN may be categorized as formulation, physio-chemical, biological, and delivery device-related factors (Figure 5). These factors may affect the absorption of medicaments from the IN route.

Accumulation in the brain may exacerbate the progression of neurodegeneration by disrupting lysosomal function, oxidative balance, mitochondrial activity, cytoskeletal integrity, microglial response, and inflammatory processes. Nanosystems need biocompatibility and biodegradability traits to effectively prevent unwanted accumulation and harm to the central nervous system (CNS), achieving swift and efficient decomposition into harmless metabolites. Substances administered intranasally are promptly cleared by the mucociliary system and drained towards the lower oral cavity or the pharynx. Furthermore, the dosage volume is limited due to the narrow dimensions of the upper nasal passages. A substance with a molecular weight below 400 Daltons can diffuse unhindered, whereas a larger molecule exceeding 1,000 Daltons may encounter obstruction within the mucus layer. These substances necessitate linkage to a ligand that enhances their availability for biological processes. Overcoming the obstacle of precisely targeting medication to its specific site of action within the CNS post-administration remains a significant challenge (69). Exploring intranasal routes for medication release emerges as a striking alternative to age-old drug administration techniques for neuropsychiatric disorder management. This technique capitalises on the nasal cavity’s unique anatomical and physiological attributes to navigate around the blood-brain barrier (BBB), hence boosting drug transit to the central nervous system (CNS). Nonetheless, despite the myriad advantages of intranasal delivery, it also encounters specific challenges that necessitate resolution to achieve optimal therapeutic efficacy.

### Advantages of intranasal drug delivery

Bypassing the blood-brain barrier (BBB): The intranasal delivery mechanism facilitates direct access to cerebral structures through the olfactory and trigeminal nerve pathways, effectively circumventing the BBB, which presents a formidable challenge for conventional drug administration methodologies such as oral and parenteral routes (70). Rapid absorption and onset of action: the substantial vascularisation and expansive surface area of the nasal cavity promote rapid drug absorption, resulting in a swifter onset of pharmacological action compared to alternative routes (71, 72). Bypassing the first-pass metabolism in the liver, drugs given intranasally improve their availability in the body and lessen systemic adverse effects. Non-invasiveness and patient compliance: the non-invasive nature of intranasal delivery significantly enhances patient adherence, particularly in chronic ailments necessitating protracted treatment regimens (73, 74). Utilizing diverse formulations like nanoparticles, gels, and emulsions could enhance drug solubility and stability, leading to the development of controlled release profiles that increase therapeutic effectiveness.

### Challenges of intranasal drug delivery

The characteristics of the nasal epithelium can restrict drug permeability, while mucociliary clearance can diminish drug retention duration, underscoring the necessity for permeation enhancers and mucoadhesive polymers to bolster delivery efficiency (71, 74). Continuous administration of nasal formulations could trigger nasal mucosa irritation, potentially harming patient adherence and the safety of the therapeutic intervention (71, 72). The formulation of effective intranasal preparations necessitates meticulous consideration of the physicochemical properties of the drug and the application of advanced technological solutions, such as nanocarriers, to ensure stability and therapeutic efficacy (75). The effectiveness of intranasal drug delivery may be contingent upon the design of the delivery apparatus and the administration technique, which must be meticulously optimized to guarantee consistent and efficacious drug delivery (76, 77).

## Nasal formulations for neuropsychiatric disorders other than nanomedicines

Caldieraro and colleagues documented the comprehensive therapeutic impact of prolonged photobiomodulation therapy (PBMT) on a patient grappling with major depression and anxiety. Over the initial 22 months, the application of single-modality i-PBM (810 nm LED, 10 Hz PW) to both nostrils resulted in a fluence of 10.65 J/cm^2^ per nostril. By progressively escalating the frequency of i-PBMT sessions, transitioning from bi-weekly sessions to daily and eventually twice daily, the patient experienced a gradual regression of anxiety symptoms, marked by an approximately three-fold reduction in anxiety symptoms (78).

The use of the IN pathway has shown promising results in facilitating the delivery of benzodiazepines to the brain for the management of sleep and anxiety disorders. Misra and Kher (77) recorded IN formulations of diazepam, lorazepam, and alprazolam based on microemulsions for managing insomnia, pointing out that the commencement and duration of sleep in male albino rats followed the hierarchy of lorazepam > alprazolam > diazepam (DA).

Ageing-related deficits in encoding or retrieving spatial memory are mitigated through the acute administration of IN-DA supplements. Previous findings highlighting the enduring favorable effects of IN-DA therapy on attention and working memory within the 8-arm Olton maze in an ADHD rat model underscore the cognitive-enhancing properties of IN-DA. These discoveries, combined with data supporting its antidepressant and antiparkinsonian properties, emphasize the promise of IN dopamine administration as a therapeutic approach to address cognitive and mood-related deficits (75).

Wink and colleagues delineated a case involving a 29-year-old woman with autism who underwent treatment with IN ketamine (20–60 mg) over 12 dosing instances across a six-week period. Subsequent to the treatment regimen, improvements were observed in mood, social interactions, flexibility, tolerance of routine changes, motivation, and concentration. Moreover, IN dopamine administration has exhibited memory-enhancing effects in an animal model of ADHD known as the naples high-excitability rat. Significantly, IN ketamine has demonstrated effectiveness in reducing the intensity of pain in cases of migraines (63).

The pivotal orchestrator of physiological function, the hypothalamic orexin (hypocretin) system, exerts influence on cognition. The IN administration of orexin-A has been demonstrated to improve cognitive function in individuals with narcolepsy and sleep deprived. Our neurochemical investigations in young and aged animals have unveiled the potential of IN orexin administration in rectifying age-related neurotransmission deficiencies (79). Deonna and team conducted an evaluation of the effectiveness, tolerance, and suitability of nasal midazolam in managing acute seizures in children, both within hospital settings and at home. Their findings indicated the efficacy of nasal midazolam in treating acute seizures sans any severe adverse effects. Furthermore, they posited that nasal midazolam could be safely employed outside hospital premises for severe epilepsies, particularly in older children, owing to its user-friendly nature for parents. Haan and colleagues compared a new midazolam HCl concentrated nasal spray with a diazepam rectal solution for prolonged human seizure management, revealing comparable efficacy and side effect profiles between the two treatments (77). Studies have illustrated that IN midazolam outperforms rectal diazepam in swiftly and effectively stopping seizure activity, and it is also more straightforward to administer than intravenous diazepam. However, there are differences in seizure cessation times across various trials (80). Research carried out by Enormous and colleagues indicated that the application of low-dose ketamine via the IN pathway leads to rapid achievement of adequate plasma levels of both ketamine and its metabolite nor-ketamine. Nonetheless, the IN delivery of ketamine did not substantially influence thermal or mechanical sensory detection and pain thresholds. Lack of randomization, blinding, or case studies are the main reasons studies evaluating other substances have limitations. Future clinical studies must establish the most effective methods for nose-to-brain drug delivery based on dosage, formulation, tools, and timing (81).

## Nanoformulations for brain disorders

In order to enhance drug delivery efficacy via the respiratory pathway, innovative drug formulations and devices must be concurrently developed. Nanoformulation emerges as a particularly auspicious approach in overcoming barriers to drug delivery. Nanocarriers play a pivotal role in augmenting drug effectiveness and present as promising formulations, thereby establishing them as essential focal points in both preclinical and clinical research environments (82). Lipid NFs have gained much traction for the transport of medicines into the brain. Their nanosize bypasses biological barriers, allowing efficient drug translocation over the BBB. Moreover, they offer numerous advantages, including controlled and specific drug delivery, minimized negative drug reactions, extended shelf life, enhanced drug absorption, and decreased drug removal to attain an optimal therapeutic drug level in the brain (55).

### Polymeric nanosuspension: drug-loaded

NFs stabilised with nonionic surfactants or lipid blends are known as polymeric nanosuspensions. The capacity to change the surface of polymeric nanosuspensions, increased drug loading, ease of fabrication, and improved pharmacokinetics are just a few advantages. Modafinil (MDF), for example, is a drug that is taken orally to treat attention deficit hyperactivity disorder (ADHD) and narcolepsy. According to the findings, MDF acted as an amorphous phase in the structure of nanoparticles. Creating NFs could be a viable way to improve oral absorption (83).

### Polymeric nanogels

Polymeric nanogels are fabricated via the cross-linking of hydrophilic or amphiphilic polymers through emulsification and subsequent solvent evaporation. The integration of ionic and non-ionic polymers results in the development of cross-linked networks, serving as the basis for nanogel creation. The utilization of various NFs, including polymeric nanogels, is anticipated to enhance the protective capabilities during the transportation of encapsulated pharmaceuticals. The primary function of polymeric nanogels has been in the conveyance of DNA, siRNA, and oligonucleotides due to their encapsulation efficiency ranging from 40 to 60%. Furthermore, nanogels have exhibited superior efficacy in delivering oligonucleotides to the brain than the spleen or liver. In the context of managing disorders like Alzheimer’s disease, Parkinson’s disease, multiple lateral sclerosis, and stroke, the combination of nanogels with spontaneously negatively charged oligonucleotides leads to the creation of a stable aqueous polyelectrolyte complex with particle sizes under 100 nm, enabling smooth traversal across the blood-brain barrier (84).

### Polymeric nanoliposomes

Polymeric nanoliposomes are vesicular structures with an internal aqueous compartment and an external lipid layer that can be single or multilamellar. With their structural architecture, nanoliposomes have increased drug encapsulation and reticuloendothelial system evasion. A synthetic phosphorylated peptide was employed to mimic a phospho-epitope of the Tau protein within a modified liposome-based amyloid vaccination. Long-term vaccination in P301L mice ameliorated symptoms and decreased tauopathy, and this endeavor is presently in Phase I. Nanoliposomes, like doxorubicin liposomal (Doxil^®^), are particularly utilized for cancer treatment (85).

### Niosomes

Niosomes are nanoscale vesicles mainly consisting of nonionic surfactants and cholesterol with a persistent bilayer structure. Niosomes are biodegradable and compatible with living organisms. Chemical stability is high, shelf life is long, toxicity is low, and production costs are low. Niosomes possess the capability to encapsulate both lipophilic and hydrophilic pharmaceutical agents, facilitating their targeted and sustained release. The influence of niosomes on the distribution of drugs within organs and their metabolic stability has been documented. Modifying the surface of niosomes to improve target selectivity for cancer medicine delivery systems has been demonstrated. Surface-modified niosomes containing olanzapine (an atypical antipsychotic treatment) showed a 3-fold increase in olanzapine concentration in the brain compared to an IN solution of the drug. Unlike nefopam hydrochloride (NF) oral solution, NF-loaded niosomes improve medication penetration via nasal mucosa and relative bioavailability (86).

### Nanospheres and nanocapsules

Nanospheres are produced through the microemulsion polymerization process, forming solid core polymeric matrices, while nanocapsules are vesicular structures that consist of an oil-filled drug compartment enclosed by a thin, biocompatible polymer. The advantages of nanospheres and nanocapsules include improved medication stability, easy surface modification, and avoiding systemic disintegration. When nanocapsules containing indomethacin are used, hippocampus cultures are protected from *in vitro* inflammation (86).

### Polymeric nanomicelles

In polymeric nanomicelles, a shell of hydrophilic polymer blocks surrounds a hydrophobic core. The shell protects and stabilizes polymeric nanomicelles from cellular interactions, while the core can encapsulate up to 30% of hydrophobic medications. However, there are no published studies on nanomicelle-mediated CNS medication delivery. Polymeric nanomicelles are expected to be effective for both *in vitro* and *in vivo* administration of DNA molecules. The hydrophobically modified glycol chitosan (HGC) nanomicelle is gaining popularity as a viable platform for chemotherapeutic drug delivery (87).

### Metal nanoparticles

Metal nanoparticles can be created with various structural and surface modifications, allowing for novel applications in magnetic separation, targeted gene and medicine administration, and, most importantly, diagnostic imaging. SERS, CT, MRI, PET, and ultrasound are modern and advanced imaging procedures requiring a contrast agent. The requirement for a contrast agent prompted the invention of nano-sized gold, silver, and magnetic iron oxide (Fe_3_O_4_) nanoparticles (88).

### Gold Nanoparticles

Gold nanoparticles (AuNPs) are widely used as nanomaterials in drug delivery and imaging. The use of AuNPs may aid in the uptake of neural signals through two distinct pathways: crossing the blood-brain barrier (BBB) and the olfactory nerves. In a research assessment, scientists efficaciously administered theragnostic polyfunctional gold-iron oxide nanoparticles that were surface-laden with miR-100 and antimiR-21 to glioblastomas (GBMs) in mice via a direct nose-to-brain transport pathway. A study revealed the development of resveratrol-loaded transferosomes and nanoemulsions, both conjugated with gold nanoparticles, for IN administration of resveratrol to the brain (89).

### Silver nanoparticles

Silver nanoparticles (AgNPs) are not typically associated with harmful effects on human skin, pulmonary, and fibroblast cells. The capacity of AgNPs to breach the blood-brain barrier (BBB) and cluster in the brain after being inhaled and ingested into the central nervous system (CNS) has been validated. Through mechanisms such as a compromised BBB, neural transmission, ocular pathways to the brain, and cellular absorption, both Ag-NPs and TiO_2_-NPs may penetrate the CNS. The interaction of nanoparticles (NPs) with glial cells might potentially initiate the release of proinflammatory cytokines, as well as the production of reactive oxygen species and nitric oxide, potentially resulting in neuroinflammation (90).

### Magnetic nanoparticles

Nanoparticles possessing magnetic characteristics are known as MNPs (magnetically nanoparticles). Magnetoporation produces temporary holes in cell membranes, as observed in the BBB endothelium, to improve medicine targeting and administration (91).

### Dendrimers

Dendrimers are a novel type of nanoparticle with a unique architecture comprising molecular hooks that may target specific cells. The dendrimer-based nanoprobe is simple to make, offers better ion imaging, and can be used with other fluorescent dyes (92).

NFs signify a substantial progression across diverse domains, encompassing pharmaceuticals, agriculture, and food fortification, attributable to their distinctive attributes and functionalities. These formulations present many benefits, including enhanced solubility, bioavailability, targeted delivery mechanisms, and diminished toxicity. They also introduce specific challenges, encompassing safety apprehensions and ecological ramifications.

### Advantages of NFs

NFs enhance drug solubility and bioavailability, thereby permitting more efficacious therapeutic interventions. They enable precise drug delivery, which can mitigate adverse effects and amplify therapeutic efficacy, particularly in managing intricate conditions such as cancer and Alzheimer’s disease (93, 94). For example, NFs can traverse the blood-brain barrier, thereby enabling the delivery of therapeutic agents to the central nervous system, a critical requirement for the treatment of neurodegenerative disorders (94, 95). Nanoparticles confer notable stability and facilitate the regulated release of pharmaceuticals, which can prolong the duration of drug action while reducing the frequency of administration (96). This aspect is particularly advantageous in therapies involving proteins and enzymes, where the preservation of structural integrity and functional activity is of importance (97). In the realm of agriculture, NFs augment the solubility and bioavailability of pesticides and fertilizers, culminating in diminished ecological impact and enhanced agricultural yields. They permit the utilization of lower concentrations of active ingredients, thereby minimizing potential hazards and optimizing the efficacy of agrochemical applications (98, 99). NFs are employed in food fortification initiatives to enhance the bioavailability and stability of micronutrients, thereby addressing nutrient deficiencies with greater efficacy than traditional methodologies. They safeguard active constituents from degradation while preserving the sensory qualities of fortified food products (100).

### Disadvantages of NFs

The diminutive dimensions and elevated reactivity of nanoparticles engender apprehensions regarding their safety and environmental implications. There exists a pressing necessity for comprehensive risk evaluations to elucidate their interactions with biological systems and ecological environments (93, 101). The potential for toxicity and long-term repercussions on human health and the ecosystem warrants meticulous scrutiny. The fabrication of NFs entails intricate processes necessitating precise particle size and distribution regulation. Such complexity may present challenges in scaling production and ensuring uniform quality (101). Furthermore, the regulatory frameworks governing NFs remain evolving, which may impede their commercialization and broader acceptance (102). In the domain of antifungal therapies, although NFs enhance drug penetration and efficacy, there exists a potential risk for the emergence of drug resistance attributable to heightened retention and exposure to the drug (102). This scenario necessitates vigilant monitoring and strategic management approaches. While NFs possess transformative potential across various sectors, their advancement and application must be reconciled with safety considerations, ecological impact, and regulatory adherence.

## Phytopharmaceuticals and bioactive molecule-loaded nanomedicine targeting nose-to-brain for neuropsychiatric disorders

Researchers respect phytomedicines because of their natural source and fewer side effects, and there has been the latest spike in inclusive curiosity in traditional medicines. Natural products have been recognized as promising neuroprotective agents in treating neurodegenerative disorders (103). On the other hand, conventional synthetic medications have been linked to unfavorable but unavoidable side effects as well as low patient compliance. Therefore, herbal remedies are progressively gaining preference over artificial chemical treatments in addressing a range of cognitive disorders (104). Traditional medicinal practices are essential in developing countries’ primary healthcare systems. Estimates indicate that approximately 80% of the global population utilizes conventional medicine (104). One hundred and seventy out of the one hundred and ninety-four member states of the World Health Organization (45) have acknowledged the utilization of traditional medicine, prompting their respective governments to seek assistance from WHO in aggregating credible evidence and data concerning these practices and products (105). The indigenous people need formal education but are well-versed in traditional medicines and how to employ them to treat various ailments. This information is passed down from generation to generation (106). Beyond symptomatic alleviation, potent and safer therapy alternatives are needed. Herbal supplements have undoubtedly been investigated for their ability to increase the effect of Western pharmaceuticals due to their multi-component composition (107).

Phytotherapy is the most common medical treatment used by people to treat illnesses of the neurological system (108). Quercetin is a flavonoid in various edible plants and is one of the most effective antioxidants discovered in plants (109). The anxiolytic and cognitive effects of quercetin, an efficient flavonol utilised as an antioxidant, were examined in male Wistar rats. The administration of quercetin to the central nervous system via IN quercetin liposomes is effective. Quercetin liposomes from cholesterol (1) and egg phosphatidylcholine (EPC) were synthesized frequently. EPC/chol at a 2:1 ratio and demonstrated anxiolytic and cognitive-enhancing properties. Quercetin liposomes are a potentially innovative method of delivering quercetin into the CNS via the IN route (110). Alzheimer’s disease (AD), Parkinson’s disease (111), traumatic brain injury (TBI), and epilepsy have all demonstrated associations with the compound known as quercetin. Quercetin can cross the BBB, making it a promising therapeutic candidate for a variety of brain disorders, including epilepsy (112). Compared to oral quercetin, both traditional and liposomal, IN quercetin liposomes had a lower dosage and a quicker effect.

Gamma-aminobutyric acid (113), functioning as an inhibitory neurotransmitter in the central nervous system (CNS), operates by interacting with chloride (Cl^−^) channels associated with receptors. The activation of GABAA receptors by neurons is involved in regulating neuronal excitability. Experimental data suggests that flavonoids, structurally resembling benzodiazepines, show antiepileptic properties through the modulation of the GABAA-Cl channel complex. Within this framework, the augmentation of neurobehavioral function is observed with the administration of curcumin-loaded nanoparticles and rutin-encapsulated chitosan nanoparticles, both possessing antioxidant properties (112). *In vitro* studies show that metal metal nanoparticles (Ag-nanoparticle) of phytoconstituents, mainly piperine, terpinolene, fargesin, piplartine and dihydrostigmasterol from fruits and roots, exhibit acetylcholinesterase inhibitory activities of the isolates and their IC_50_ values were determined via a colourimetric assay (114).

Nutraceuticals are becoming more widely used to complement modern Western treatment worldwide. Neuro-nutraceuticals are therapeutic foods with phytochemicals that can benefit the young and old brain. Their physiological activities may 1 day be used to boost cognitive performance or to treat devastating neuropsychiatric and neurodegenerative diseases (107). Several clinical studies have demonstrated the efficacy of herbal medicinal plants in the treatment of CNS illnesses such as depression, anxiety, insomnia, Parkinson’s disease, obsessive-compulsive disorder, and poor cognition (Table 2) (107, 115). For Alzheimer’s disease therapy, huperzine A (HupA), found in the botanical sample *Huperzia serrata*, serves as a reversible inhibitor of acetylcholinesterase (116) and has been proven to enhance memory in various behavioral animal paradigms. The application of HupA-loaded PLGA NPs co-modified with Lf and TMC (Lf-TMC NPs) illustrates effectiveness in facilitating the IN delivery of HupA to the brain. The emulsion–solvent evaporation approach made mucoadhesive bifunctional HupA Lf-TMC NPs. The improved HupA Lf-TMC NPs had a suitable particle size, polydispersity index, and a high EE and positive potential. *Ex vivo* drug discharge and cell survival experiments employing the 16HBE cell line demonstrated that the produced NPs for IN delivery had regulated drug release and were safe (117). Regarding mental function improvement, folic acid-niosomes obtained from *Asparagus racemosus* had a high EE and a more excellent *in vitro* drug release of 64.2 per cent after 12 h, making them an optimal formulation. After 6 h, the nasal cavity is responsible for 48.15 per cent of the drug’s absorption (118). Eight phytochemicals with drug-like properties (referred to as DPCs) have been documented as a plausible avenue for regulating PCOS and its associated comorbidities within *T. purpurea.* The phytochemicals that showed a strong binding affinity for their protein targets were also compared to presently licensed medicines in DrugBank (119).

**Table 2:** List of bioactive molecules loaded-nanoformulation administered intranasally for the treatment of NPDs.

S. No.	Disease	Nanoformulation	Loading capacity	Release	Plant details	Bioactive molecule	Remark	References
1.	Anxiety	Liposomes and rutin-encapsulated chitosan nanoparticles	39.48% ± 3.16%	80.23%, 1 h	*Jasminum sambac* (Oleaceae)	Quercetin, rutin, beta-sitosterol etc.	Better permeability than oral administration	(109, 110, 141)
2.	Cognitive impairment and mental function	Niosomes	69.42%	64.2% at 12 h	*Asparagus racemosus* (Liliaceae)	Folic acid, polysaccharide, recemosol etc.	High EE and more excellent *in vitro* drug release	(118)
		HupA-loaded PLGA NPs	2.86 ± 0.6%	72.1% at 48 h	Club moss *Huperzia serrata*	Huperzine A	Good particle size and polydispersity index, plus a high EE and a positive potential	(113)

## Synthetic molecules derived nanomedicines targeting nose-to-brain for neuropsychiatric disorders

Synthetic drugs are chemically modified molecules that exhibit pharmacological effects after binding to the target site. Despite increased affinity and efficacy, synthetic polymers have drawbacks such as high cost, limited availability, safety concerns, environmental hazards, and accumulation in the body over time. Anxiety disorders are treated with pharmaceuticals (such as antidepressants, anti-anxiety meds, and-adrenergic blockers), particular types of psychotherapy (such as cognitive-behavioral therapy), or both. Anxiety disorders are routinely treated with anxiolytic, myorelaxing, sedative, and anticonvulsant tranquillizers. However, several tranquillizers are being used to treat a wide range of medical disorders. Buspirone, meprobamate, benactin, and other similar medicines are examples. Buspirone hydrochloride was incorporated into polymeric thiolated chitosan nanoparticles for IN delivery (113). However, when buspirone hydrochloride was employed in the composition of the carbopol nanovesicular gel, its bioavailability increased by 3.26 times compared to a pure medication nasal solution (8). Antidepressants with various modes of action that affect monoamine neurotransmission in the brain are commonly used to treat depression. Monoamine oxidases A and B (MAO-A and B) in the CNS are important in intracellular neurotransmitter metabolism. The use of non-selective first-generation MAO inhibitors as neuropsychiatric treatments caused a variety of undesirable side effects, including hypertensive crises and cheese reactions (120).

The research conducted by Singh et al. (121) indicated that thiolated chitosan nanoparticles enhanced the delivery of selegiline (a MAO-B inhibitor) through the nasal route and manifested an antidepressant-like effect in rats (8). In rats that were administered injections, SSRIs like paroxetine nanoemulsion and fluoxetine in an ion-sensitive *in situ* nasal gel showed significant variations in locomotor activity and immobility compared to the control groups. Upon comparison between the IN administration of nanoparticles loaded with venlafaxine hydrochloride (SARIs) and oral administration of equivalent doses, a more pronounced effect was observed *in vivo*. In addition, the IN administration of agomelatine-loaded poly-lactic-co-glycolic acid nanoparticles also illustrated antidepressant-like effects in rats (8).

Albumin nanoparticles with cyclodextrins were also synthesized for nasal administration and advancement of therapeutic characteristics (36). Nowadays, siRNA gene therapy is a viable treatment option for a variety of mental illnesses. Beta-secretase 1 (BACE1) siRNA was delivered to the brain using solid lipid nanoparticles with and without chitosan. The peptide RVG-9R, originating from the glycoprotein of the rabies virus, was incorporated into these nanoparticles, leading to enhanced transcellular distribution of nanosystems to the brain following IN administration (8).

Chlorpromazine, the first medicine for the treatment of schizophrenia, was initially used in 1952. Still, researchers began working on long-acting compounds combining an antipsychotic agent with a fat-soluble solution in the 1960s. Because of the poor absorption of sarcosine into the brain, these clinical experiments with sarcosine have prompted the creation of specific GlyT-1 inhibitors. The outcomes of a phase II proof-of-concept trial involving 320 patients utilizing Roche’s investigational GlyT-1 inhibitor RG1678 were disclosed today. Within individuals diagnosed with schizophrenia, RG1678 demonstrates a pronounced and clinically significant influence (122). Medications for psychosis, which reduce the heightened dopaminergic signaling in patients’ brains, are the most frequently prescribed treatments for this disorder (123). Second-generation atypical antipsychotic agents, distinguished by a more limited array of adverse effects in comparison to the initial generation, are utilized in the treatment of bipolar disorder alongside lithium and antiepileptic medications. The IN delivery of poly (lactic-co-glycolic acid) nanoparticles has been shown to improve the efficiency of olanzapine (PLGA) nanoparticle transport to the brain. Following the IN administration of olanzapine-SLNs, the relative bioavailability of olanzapine within the brain was elevated by a factor of 23. The IN administration of poly-caprolactone nanoparticles resulted in a twofold increase in the distribution of aripiprazole in the rat brain compared to aripiprazole nanoparticles administered intravenously. When the IN administration of risperidone-loaded mucoadhesive nanoemulsion was compared to the intravenous delivery of risperidone nanoemulsion in Swiss albino rats, it was observed that the IN method led to enhanced efficacy, improved brain targeting, and increased drug transport to the brain. During the IN injection of quetiapine, the bioavailability of the mucoadhesive microemulsion increased. Some researchers produced sublingual film, intranasal, and injectable versions of azenapine to increase its bioavailability (8).

NLC-based formulations resulted in greater drug diffusion across sheep nasal mucosa. In comparison to the administration of carbamazepine (CBZ) through oral dispersion and CBZ *in-situ* gel via the nasal route, the utilization of a nanolipid carrier (NLC) in a rat model of maximal electroshock (MES) displayed enhanced *in vivo* anticonvulsant effectiveness. An IN formulation utilizing NLCs for CBZ delivery holds promise in improving the drug’s anticonvulsant efficacy (124). The IN delivery of NLCs loaded with valproic acid (VPA) resulted in a notably higher brain plasma concentration ratio than the control group, as evidenced by drug levels determined in both brain tissue and plasma. Finally, IN delivery of VPA NLCs gave superior protection against MES seizure than oral administration (125). The dosage of oxcarbazepine (OXC) that proved effective in suppressing epileptic seizures in rats following IN injections was 0.5 mg/kg (1 dose every 20 min for 1 h), as validated by CSF bioavailability. After a 16-day treatment period, the immunohistochemical evaluations (anti-neurofilament, anti-beta tubulin, and anti-caspase3) indicated that OXC PLGA NPs exhibited a neuroprotective influence. These affirmative results illustrated the potential for establishing a non-invasive nose-to-brain delivery mechanism for OXC in epilepsy therapy (126). Developing specific and selective MAO inhibitors, such as moclobemide and toloxatone, has enhanced their effectiveness as therapeutic agents in monotherapy and adjunctive treatment. While MAO inhibitor-containing botanicals are commonly utilized in the development of potent synthetic drugs, they also serve as secure and efficient alternatives to existing synthetic treatments for neurodegenerative conditions like depression, Parkinson’s disease, and Alzheimer’s disease (116).

Despite the range of treatment options available, health professionals dealing with psychiatric patients continue to be concerned about depression, anxiety, and treatment. The effectiveness of the antidepressant medication is determined by the drug’s concentration in the brain. Traditional oral and parenteral therapy is limited because the medicine must penetrate the BBB. A few studies have been done to develop nanomedicine for treating NPDs (Table 3). Research indicates that the IN application of nanotherapy, where medications are enclosed within nanoparticles, represents the optimal approach for transporting a diverse array of drugs to the brain.

**Table 3:** List of synthetic drugs loaded nanoformulation administered intranasally for the treatment of brain disorders.

S. No.	Disorder	Current drug	Nanoformulations	Loading efficiency	Release	Effects	References
1.	Anxiety	Buspirone HCL Ritanserin Fluoxetine Fluvoxamine Sertraline	BUH thiolated chitosan NPs BUH nanovesicles	70.1% ± 1.2%	Up to 72 h Up to 48 h	The brain concentration achieved a higher 3.26 times increase of BUH bioavailability	(142)
2.	Depression	Agomelatine Duloxetine Folic acid Selegiline Vanlafaxine	AgomelatinePLGA NPs Duloxetine nanostructured lipid carriers Folic acid niosomes Selegiline Hydrochloride thiolated chitosan NPs Venlafaxine alginate NPs	10 to 40% 76.21% 69.42% 86.2% High loading	Up to 30 days 54% drug release over 8 h 64.2% at the end of 12 h 82.529% over 28 h 80% released over 96 h	Prominent antidepressant activity Better brain targeting & decreased side effects 48.15% of the drug is absorbed through the nasal cavity Attenuation of the oxidative stress and restoring the mitochondrial complex activity The more excellent brain/blood ratios	(8)
3.	Autism spectrum disorders	Risperidone Aripiprazole Lithium Haloperidol Clozapine Fluoxetine Methylphenidate	Oxytocin nasal spray Dexmedetomidine and Midazolam	82%	80% released over 72 h	Improvement in social abilities Sedation rate increases	(143–145)
4.	ADHD	Desipramine Imipramine Nortriptyline Dextroamphetamine Methamphetamine Methylphenidate Pemoline	Dopamine hydrochloride	35%	100% release over five days	10 μL of a gel composed of a viscous castor oil mixture	(43, 146–148)
5.	Schizophrenia and bipolar disorders	Aripiprazole Haloperidol Lurasidone Olanzapine Paliperidone Risperidone Quetiapine fumarate	Asenapine maleate Chitosan coated nanostructured lipid carrier Haloperidol Lectin-functionalized, PEG-PLGA NPs Lurasidone Nanolipid carrier Olanzapine CS NP PLGA NPs Nanocubic vesicles (NCV) liposomes	78.62% 84% 6.14% and an entrapment efficiency of 77% Entrapment efficiency of 72.42% and a drug loading capacity of 26.04% Encapsulation efficacy 78.53 to 96.12% Encapsulation efficacy 85%	98.88% over 36 h 70% over 24 h Therapeutic levels over extended periods Sustained release Sustained release over 30 days Sustained release up to 8 h	2.3 and 4-fold higher systemic and brain bioavailability Increase of the haloperidol concentrations by 1.5-3-fold in brain tissue A 2-fold increase of the drug concentration in the brain Improve the systemic absorption Increase 6.35 and 10.86-fold-uptake than pure olanzapine 37.9% absolute bioavailability and 100% brain targeting efficiency	(8, 149)

Compared to other drug carriers, nanodiamond (ND) particles promote the adsorption of different functional groups and drug molecules more significantly, with electrostatic interaction potential generated by the surface functionalities and the large surface area about their volume. To acquire absorption into the subject and transportation to the targeted sites, the ND particles-based formulation can be administered in any preferred way or by any desired route of administration (e.g., the BBB). All options are intravenous, oral, subcutaneous, intramuscular, intranasal, pulmonary, or rectal administration. ND, nanometer-sized crystalline carbon particles (e.g., <10 nm), have shown a remarkable capacity to tolerate surface changes. ND particles have shown effective treatments against different psychological and neuropsychiatric illnesses connected to AIDS in the CNS (all instances of neuro-AIDS) (127). The polyelectrolyte complexes of the discussed invention can take the shape of a particle, such as a microparticle (less than 1 mm in diameter) or a nanoparticle (less than 1 mm in diameter). After IN delivery, nano-SODl showed increased absorption in the entire brain, olfactory bulb, and hypothalamus but no change in serum or hippocampus compared to native SODl. At 5 and 20 min after IN treatment, crosslinked nano-SODl showed better brain absorption than native SODl and PEGylated SODl (sigma product). Similarly, compared to saline-treated mice with animals treated with polypeptide nanoparticles such as BDNF, nano-BDNF formulation exhibited considerably less tissue loss (128).

## Clinically available drugs for neuropsychiatric disorders

In the year 2019, an intranasal formulation of esketamine, branded as SPRAVATO^®^, received approval from both the FDA and the European Medical Agency (EMA) as a swift-acting antidepressant intended for the management of major depressive disorder (MDD), subsequent to the favorable outcomes observed in clinical trials. These trials demonstrated prompt alleviation of depressive symptoms, including suicidal ideation, enhanced mood, prolonged efficacy, and a commendable safety profile (129). Subsequently, the prospective application of intranasal esketamine for bipolar disorder and MDD accompanied by comorbidities, such as post-traumatic stress disorder and psychosis, has been investigated in patient cohorts, yielding similarly positive results (129). The endorsement of intranasal esketamine for clinical application has catalyzed investigational efforts regarding this method of administration for central nervous system (CNS) drug delivery, particularly in scenarios where pharmacotherapeutic agents are rapidly metabolized in the periphery, exhibit difficulty in traversing the blood-brain barrier (BBB), or are linked to systemic adverse effects.

## Future perspectives

Despite the many advantages, it utilizes NF through IN delivery, which necessitates further scrutiny to advance the treatment of neuropsychiatric disorders. The greater the availability of various types of NF, the more options for the target site. Furthermore, more effective treatments can be provided. Before that may happen, the efficacy of NFs administered intranasally must first be evaluated. A thorough characterization of the various NFs, extensive *in vitro* and *in vivo* evaluations, and biocompatibility and toxicity assessments should be done. Formulation scientists can work toward producing effective and safe NFs for treating neuropsychiatric conditions with a targeted research strategy.

## Conclusion

There has been a lengthy list of drugs created for various CNS illnesses that have been rejected due to their inability to cross the BBB. Despite the advancements seen in the development of drug treatments for central nervous system (CNS) issues, a multitude of therapeutic compounds have been shown to lack effectiveness because they have trouble getting past the blood-brain barrier (BBB). Neuropsychiatric disorders (NPDs) necessitate prolonged therapeutic interventions, thereby requiring sustained and reliable drug administration. The field of nanomedicine presents a compelling solution by utilizing nano-carrier systems that facilitate extended, targeted, and regulated drug release, effectively addressing fundamental challenges such as bioavailability, BBB translocation, and accurate delivery to the affected cerebral regions. Recent advancements in nanotechnology research and associated patents have commenced tackling these issues; however, the intranasal (IN) delivery of nanostructures (NFs) for managing NPDs continues to be insufficiently investigated. Our investigation underscores the transformative capabilities of nanocarriers in augmenting the therapeutic efficacy of pharmaceutical agents for NPDs. By surmounting the conventional obstacles to CNS drug delivery, we contribute to establishing a novel paradigm that amalgamates natural and synthetic compounds into more effective, patient-centric delivery mechanisms. These delivery systems enhance patient adherence and present a promising pathway for addressing unmet medical requirements in neuropsychiatric treatment. As nanomedicine continues to evolve, our research forms the basis for trailblazing treatment strategies that might revolutionize drug delivery practices and significantly affect human health and holistic well-being.
